# Supplementary material for: Curcumin and Resveratrol Regulate Intestinal Bacteria and Alleviate Intestinal Inflammation in Weaned Piglets
Source: Molecules. 2019 Mar 28;24(7):1220. doi: 10.3390/molecules24071220 (PMC6479679; doi:10.3390/molecules24071220)
Supplement: Supplementary file 1 [file molecules-24-01220-s001.pdf]

molecules

Supplementary Materials

Table S1. Composition and nutrient levels of the basal diet

Ingredients	%
Corn	65.8
Soybean meal	24
Soybean oil	2.5
Fish meal	1.5
Limestone	0.85
$CaHPO_4$	1
Salt	0.35
Premix[a]	4
Nutrient	
Digestive energy(MJ/kg)[b]	1525
Protein (%)[b]	18.5
Calcium (%)[b]	0.70
Phosphorus (%)[b]	0.55
Lysine (%)[b]	1.32
Crude fibre (%)[b]	3.25

[a] Supplied per kilogram of diet: vitamin A, 2200 IU; vitamin D-3,220 IU; vitamin E, 16 IU; vitamin K-3, 0.5 mg; biotin, 0.05 mg; folic acid, 0.3 mg; pantothenic acid, 10 mg; niacin, 15 mg; vitamin B2, 3.6 mg; vitamin Bl, 1.0 mg; vitamin B6, 1.5 mg; Cu, 6 mg; Fe, 100 mg; Zn 110 mg; Mn, 4 mg; Se, 0.3 mg; I, 0.14 mg

[b] calculated value

Table S2 Primer sequences used in quantitative real-time PCR assays

Gene	Accession no.	Primer, 5′–3′
IL-1β	NM_214055.1	AAG TGG TGT TCT GCA TGA GC
		AAG ACG GGC TTT TGT TCT GC
TNF-α	X57321.1	TCC ACC AAC GTT TTC CTC AC
		TGC CCA GAT TCA GCA AAG TC
IL-10	NM_214041.1	TCA AAC GAA GGA CCA GAT GGG
		ACG GCC TTG CTC TTG TTT TC
β-actin	XM_003124280.4	CGG CAT CCA CGA AAC TAC CT
		ACT CCT GCT TGC TGA TCC AC
TLR4	EU716413.1	ACA GGT ATC CCA GAG GGC AT
		AGC TGC TTC TGG TCC TTG AC
TLR2	NM_213761.1	CGT GTG CTA TGA CGC TTT CG
		GGA AGT GGG AGA AGT CCA GC

IL-1β, Interleukin-1 alpha; *TNF-β*, Tumor necrosis factor-beta; *IL-10*, Interleukin-10; β-actin, Beta-acin; *TLR4*, Toll like receptor 4; *TLR2*, Toll like receptor 2.

Table S3. Primers used for intestinal bacteria

**Items**	**Primer sequence (5′-3′)**	**Annealing temperature (℃)**	**Reference**
Total bacteria	ACTCCTACGGGAGGCAGCAG	60	[59, 61]
	ATTACCGCGGCTGCTGG		
Escherichia coli	CATGCCGCGTGTATGAAGAA	60	[61]
	CGGGTAACGTCAATGAGCAAA		
Bifidobacterium	CGCGTCCGGTGTGAAAG	60	[61]
	CTTCCCGATATCTACACATTCCA		
Lactobacillus	GAGGCAGCAGTAGGGAATCTTC	60	[61]
	CAACAGTTACTCTGACACCCGTTCTTC		
Enterococcus	CCCTTATTGTTAGTTGCCATCATT	60	[60, 61]
	ACT CGTTGTACTTCCCATTGT		
Clostridium	GAGTTTGATCMTGGCTCAG	60	[60, 61]
	CCCTTTACACCCAGTAA		
